# Supplementary material for: Content-rich biological network constructed by mining PubMed abstracts
Source: BMC Bioinformatics. 2004 Oct 8;5:147. doi: 10.1186/1471-2105-5-147 (PMC528731; doi:10.1186/1471-2105-5-147)
Supplement: Additional File 2 — The original results of the above study (non-essential files are deleted to keep the file size under the limit set by BMC bioinformatics). [file 1471-2105-5-147-S2.bz2 › chilibotAdditionalFile2/dip10/318ID10583954E2439/html/GSN_ACTB.html]

 


 **GSN** and **ACTB** 
  
Found 10 abstracts in PubMed, retrieved 10.  
 

 What does Google say? 
 PDF only 
| .edu only 

---

**Interactive relationship** (e.g. stimulation, inhibition, etc)

**Neutral relationship**- The mutant beta actin  [ **ACTB** ]  binds to ADF  [ **GSN** ]  with about the same affinity as normal actin.  Ref: 9079652 J Biol Chem, 1997
- Myoblasts, transfected with a human gene encoding a beta actin  [ **ACTB** ]  point mutation, down regulate expression of actin depolymerizing factor  [ **GSN** ]  ADF  [ **GSN** ]  and its mRNA.  Ref: 9079652 J Biol Chem, 1997
- The three dimensional structures of myosin subfragment 1 S1, gelsolin  [ **GSN** ]  segment 1 complexed with alpha actin, villin fragment 14T, Acanthamoeba profilin I, and bovine profilin complexed with beta actin  [ **ACTB** ]  were completed last year.  Ref: 8167031 Curr Opin Cell Biol, 1994
- Gelsolin  [ **GSN** ]  was cross linked to F and G actin by the zero length cross linker 1 ethyl 3 3 dimethylamino propyl carbodiimide EDC, generating a covalent 130K binary complex actin1 gelsolin1 followed by a covalent 180K ternary complex actin2  [ **ACTB** ]  gelsolin1.  Ref: 3017407 Biochemistry, 1986

**Non-interactive relationship** (e.g. studied together, co-existance, homology, etc.)

- Phosphorylation of gelsolin  [ **GSN** ]  in the gelsolin  [ **GSN** ]  actin2  [ **ACTB** ]  complex was inhibited by 90%.  Ref: 10210201 Protein Sci, 1999
- beta Actin  [ **ACTB** ]  and gelsolin  [ **GSN** ]  associated transiently with beads followed by LAMP 2 and cathepsin B.  Ref: 10945978 J Biol Chem, 2000
